# Deep learning-based NLP data pipeline for EHR-scanned document information extraction

**DOI:** 10.1093/jamiaopen/ooac045

**Published:** 2022-06-11

**Authors:** Enshuo Hsu, Ioannis Malagaris, Yong-Fang Kuo, Rizwana Sultana, Kirk Roberts

**Affiliations:** Department of Biostatistics and Data Science, University of Texas Medical Branch, Galveston, Texas, USA; School of Biomedical Informatics, University of Texas Health Science Center at Houston, Houston, Texas, USA; Center for Outcomes Research, Houston Methodist, Houston, Texas, USA; Department of Biostatistics and Data Science, University of Texas Medical Branch, Galveston, Texas, USA; Department of Biostatistics and Data Science, University of Texas Medical Branch, Galveston, Texas, USA; Division of Pulmonary, Critical Care and Sleep Medicine, Department of Internal Medicine, University of Texas Medical Branch, Galveston, Texas, USA; School of Biomedical Informatics, University of Texas Health Science Center at Houston, Houston, Texas, USA

**Keywords:** scanned document, optical character recognition, natural language processing, electronic health records, polysomnography

## Abstract

**Objective:**

Scanned documents in electronic health records (EHR) have been a challenge for decades, and are expected to stay in the foreseeable future. Current approaches for processing include image preprocessing, optical character recognition (OCR), and natural language processing (NLP). However, there is limited work evaluating the interaction of image preprocessing methods, NLP models, and document layout.

**Materials and Methods:**

We evaluated 2 key indicators for sleep apnea, Apnea hypopnea index (AHI) and oxygen saturation (SaO_2_), from 955 scanned sleep study reports. Image preprocessing methods include gray-scaling, dilating, eroding, and contrast. OCR was implemented with Tesseract. Seven traditional machine learning models and 3 deep learning models were evaluated. We also evaluated combinations of image preprocessing methods, and 2 deep learning architectures (with and without structured input providing document layout information), with the goal of optimizing end-to-end performance.

**Results:**

Our proposed method using ClinicalBERT reached an AUROC of 0.9743 and document accuracy of 94.76% for AHI, and an AUROC of 0.9523 and document accuracy of 91.61% for SaO_2_.

**Discussion:**

There are multiple, inter-related steps to extract meaningful information from scanned reports. While it would be infeasible to experiment with all possible option combinations, we experimented with several of the most critical steps for information extraction, including image processing and NLP. Given that scanned documents will likely be part of healthcare for years to come, it is critical to develop NLP systems to extract key information from this data.

**Conclusion:**

We demonstrated the proper use of image preprocessing and document layout could be beneficial to scanned document processing.

## INTRODUCTION

Scanned documents in electronic health records (EHR) have long been reported as a problem.^1^ Generally, these documents are the result of faxed medical records, paper-based documents, and external laboratory reports. Despite the efforts in technical solutions,^2^^,^^3^ it seems clear that for the foreseeable future, scanned documents in the EHR will continue to play a prevalent part in our medical record ecosystem. It is thus critical to have informatics approaches to process information in scanned documents. Common approaches to handling scanned documents include image preprocessing, optical character recognition (OCR), and text mining. Prior publications have reported promising results of adopting aspects of this workflow for real-world challenges.^3–5^ However, there is limited work evaluating: (1) the choice of image preprocessing methods, (2) the selection of NLP models, and (3) the utilization of document layout. The impact of each element and the interplay between them remain unexplored. Furthermore, as deep learning-based natural language processing (NLP) progresses and new state-of-the-art language models based on Transformers^6^ are introduced, scanned document information extraction studies have not kept pace with those advanced methods. Therefore, in this study, we propose the first data pipeline adopting Transformer-based NLP models for scanned document information extraction and the first work that evaluates the impact of image preprocessing methods, NLP model selection, and document layout utilization in scanned document processing for EHRs. Focusing on a use case, our data pipeline extracts 2 key measurements for sleep apnea: Apnea hypopnea index (AHI) and oxygen saturation (SaO_2_), from scanned sleep study reports. AHI, defined as the average count of apnea and hypopnea per hour is the gold standard for sleep apnea diagnosis and severity categorization while SaO_2_ provides additional clinical information regarding intervention.^7^ The insights of this study are summarized based on the evaluation of 6 image preprocessing methods, 7 machine learning-based bag-of-word models, 3 deep learning-based sequence models, and the document layout for modeling.

## RELATED WORK

### Information extraction from scanned documents in EHRs

Several scanned document processing studies focus on pathology and imaging reports containing important clinical concepts and numeric values which are embedded in free-text narratives or nonstandardized formats.^3^^,^^4^^,^^8^ Sources of scanned documents include paper-based case report forms and outpatient referral forms which are created during hospital workflow that involves handwriting.^5^^,^^9^ Documents are often scanned and stored as images in Portable Document Format (PDF). Current approaches for processing scanned EHR documents often involve 2 continuous steps: OCR and text mining. OCR extracts words from scanned images and converts them into machine-readable text, and text mining further extracts clinically relevant information. A wide variety of OCR engines have been used including Adobe Acrobat Pro, FormScanner, and Tesseract. Most studies use rule-based algorithms while state-of-the-art deep learning-based NLP models are rarely attempted. Besides the 2 main steps, image preprocessing often improves scanned image quality.^5^^,^^10^ Image segmentation isolates text components from the background. Gray-scaling reduces the computational burden. Erosion regularizes the text mapping. Thresholding separates information from its background.^11^ Those have been adopted occasionally in individual studies to improve OCR outputs. However, there is no systematic evaluation of these methods and there is limited guidance for image preprocessing and text mining.

### Information extraction from scanned documents in nonclinical fields

Handwriting recognition, scanned receipt information extraction, and automatic cheque processing are some applications of scanned document processing.^11^^,^^12^ In scanned receipt recognition, a recent study developed a processing pipeline that utilized deep learning: the Connectionist Text Proposal Network (CTPN) for text detection and the Attention-based Encoder-Decoder (AED) for text recognition.^13^ In cheques recognition, a recent publication finds a 2D Convolution Neural Network following image preprocessing using Otsu thresholding could achieve a 95.71% accuracy from a pool of sample cheques.^14^

### OCR techniques

The common OCR processes start with *segmentation* in which word lines, words, and characters are isolated from the image background. Characters are represented as matrices of pixels followed by the *normalization* that minimizes matrix size and reduces noise. Then, for each pixel matrix, *feature extraction* creates a feature vector to represent it. Statistical or machine learning *classifiers* are used to categorize feature vectors to match existing characters and thus output machine-readable words.^11^^,^^15^ Recent OCR engines adopted deep learning architectures, including Long short-term memory (LSTM) for character classification.^16^ Unlike other fields, there is limited work on the development and evaluation of OCR methods for the medical domain. An earlier study evaluated 3 general domain OCR engines: tesseract 3.0, Nuance, and LEADTOOLS on hand-written forms in EHR.^1^ While recent studies focus on post-OCR spelling corrections.^4^^,^^10^^,^^17^

In summary, it is well-acknowledged that scanned documents still pose technical challenges for EHRs, as well as scientific challenges for how best to extract information from them. However, what is missing is an understanding of the interplay of how this information can be extracted, especially using modern machine learning-based NLP techniques. This is the gap this paper seeks to fill.

## METHODS

### Data source

We utilized manually reviewed sleep study reports from an existing study at the University of Texas Medical Branch (UTMB) (IRB# 19-0189). In that prior study, the UTMB EHR (Epic Systems) was queried for data from June 1, 2015 to May 31, 2018. A total of 3720 patients who had at least 2 outpatient visits to pulmonary clinics or primary care providers (PCP), were at least 18 years old, had at least 1 sleep disorder diagnosis code, and had BMI on record were included. The study randomly sampled 1200 patients (800 from pulmonary clinics and 400 from PCP) for manual chart review, performed by a group of 4 sleep medicine specialists. Among the sampled patients, the AHI and SaO_2_ (minimum SaO_2_) values from 990 sleep study reports were found and recorded in a separate sheet. Some numeric values were rounded to integers during recording. Each report was only reviewed once by 1 of the 4 reviewers and no inter-reviewer agreement was evaluated. Our current study utilized the 990 reviewed reports and was approved by the institutional review board (IRB# 20-0266). We recovered the original numeric values by looking up the scanned reports. We also excluded 35 reports without complete AHI and SaO_2_ records. Our final dataset contains 2988 scanned PDF images (from 955 unique reports).

### Image preprocessing

**Figure 1.**
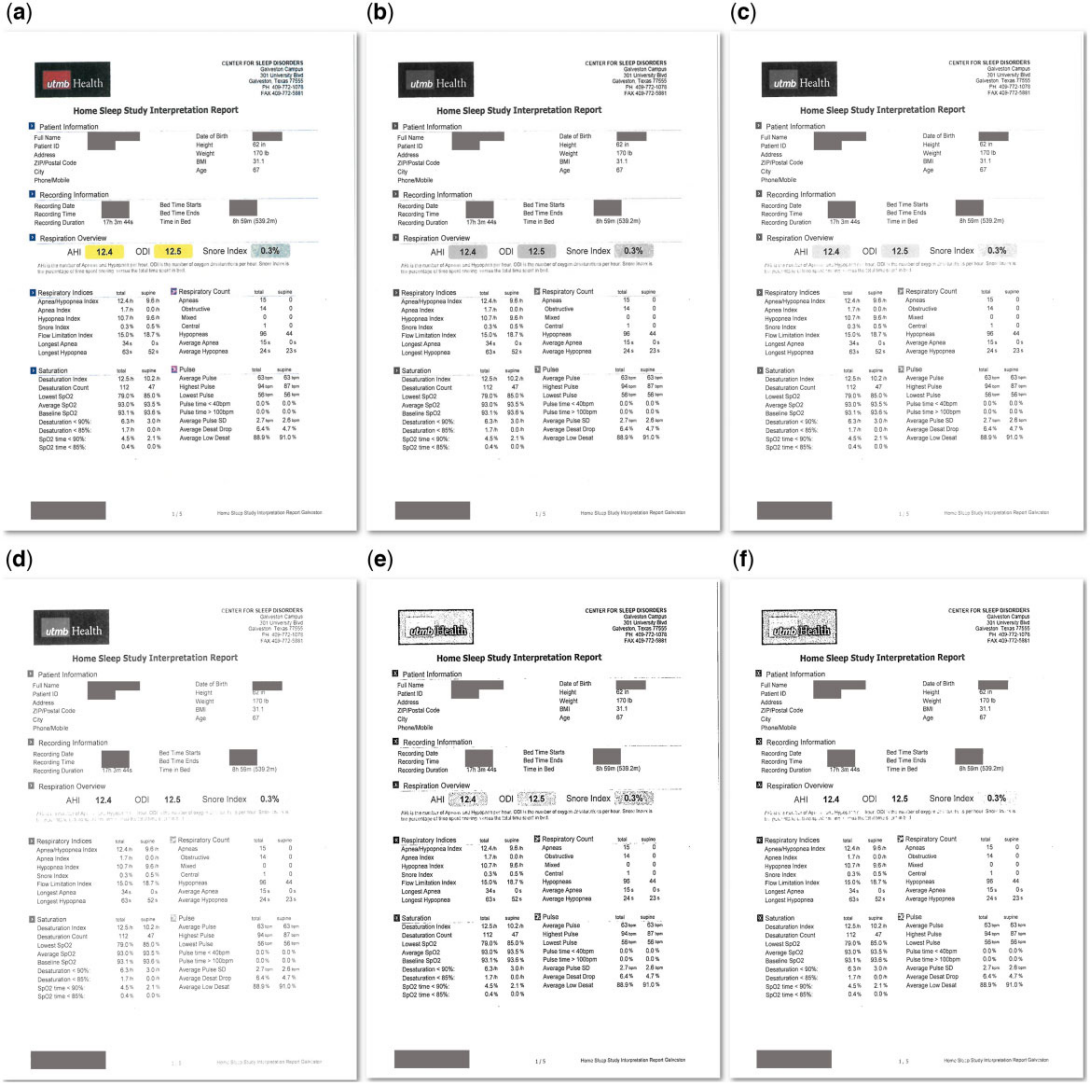
Scanned document images after image preprocessing. (A) The original scanned image. (B) The gray-scaled image. (C) The image with 20% increased contrast. (D) The image with 60% increased contrast. (E) The image with dilation and erosion and 20% increased contrast. (F) the image with dilation and erosion and 60% increased contrast.

We extract images pages from the PDF files followed by image preprocessing using the Open Source Computer Vision Library (OpenCV, version 4.5.2).^18^ We first convert the 3-channel color images to 1-channel gray-scale to reduce computation complexity, then dilate and erode each character by 1 iteration of transformation.^10^^,^^19^ The dilation process shrinks objects (characters) and results in the removal of small noise dots, while the erosion process converts the image back to the original scale. Finally, we increased the contrast by 20% thus background noises caused by scanning were further removed (Figure 1).^20^

### Optical character recognition

**Figure 2.**
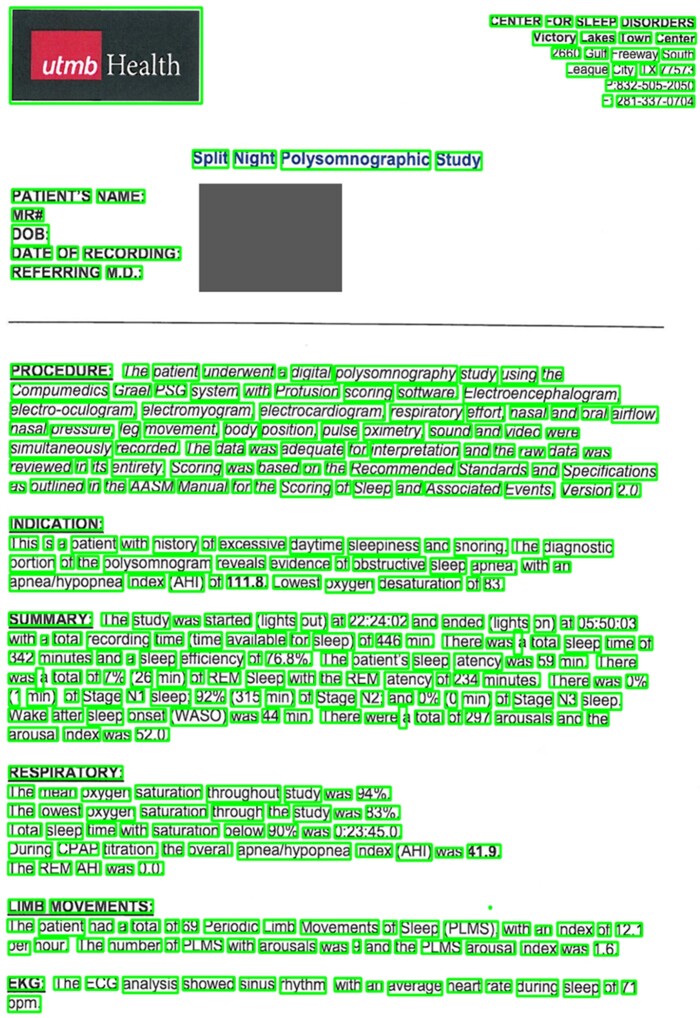
Output of OCR for visual inspection.

We apply Tesseract OCR (version 4.0.0)^16^ via *pytesseract*^21^ to locate and extract machine-readable text from the preprocessed images. The output for each image is a mapping of extracted words and positions in pixels. We performed a data quality visual inspection by programmatically drawing outlines of each word onto the original images using the positions with OpenCV (Figure 2).

### Deidentification

To ensure the confidentiality of patient information, we deidentified the output from OCR. We queried the EHR to create a lookup table with report ID, patient names, and medical record numbers. Searching among the OCR-extracted texts in each report, the matched patient name and medical record number were masked with placeholders (“[PATNAME],” “[MRN]”). To exclude the date of birth and procedure dates, any output words with date formats (“XX/XX/XXXX”) were replaced by placeholders (“[DATE]”).

### Text segmentation

Each sleep study report on average has 3 pages with multiple paragraphs of free text. Candidate words for AHI and SaO_2_ values are identified using a regular expression for words that match “[0-9.,%]+”. For each numeric value, a segment of 10 words on each side of the candidate (21 words total) is used for context. Examples are shown in Table 1.

**Table 1.** Analytical data for text classification

Left	Top	Width	Height	Page	Numeric value	Segment	Label
1048	385	111	50	1	19.5	The Medicare scoring rule. The total APNEA/HYPOPNEA INDEX (AHI) was **19.5.** The patient also had 0 respiratory event related arousals (RERA)	AHI
231	558	76	23	1	87.0	Versus a non-REM AHI of 15.1. The lowest desaturation was **87**, with a mean value of 95. The patient had a	SaO_2_
735	388	61	26	1	26.0	Hypopneas, 120 met the AASM Version 2 scoring rule, while **26** met the Medicare scoring rule. The total APNEA/HYPOPNEA INDEX (AHI)	Other

*Note*: The column “Left” and “Top” are the coordinate of pixels for the top-left corner of the word regions. The column “Width” and “Height” are the width and height in pixels of the word regions. The column “Page” indicates from which page of the document the numeric value was extracted. The column “Numeric value” is the floating point representation of the numeric value. The column “Segment” holds the free text segment of 21 words. We label the numeric value in bold. The column “Label” was derived from manual chart review and was used as the label for the supervised learning classifiers.

### Text classification

At this point, the information extraction problem can be cast into a 3-way classification task: whether the candidate numeric value is an AHI value, a SaO_2_ value, or neither. Each instance has a set of position indicators obtained from OCR, the page number from which the numeric value was extracted, a floating-point representation of the numeric value, and a segment of 21 words. Our human review did not include information on positions where the AHI and SaO_2_ values were found. We assigned labels by matching the recorded AHI and SaO_2_ numbers to each of the numeric values in the document. Therefore, as a limitation, we cannot rule out false positives if some other numeric values in the same report happened to be the same number as the AHI or SaO_2_, though we suspect this to be quite rare.

In our main experiment, we construct and train 2 types of NLP models: bag-of-word models and deep learning-based sequence models.

### Bag-of-word models

A bag-of-word model that only considers term frequency is the traditional approach for text classification. We remove English stopwords and lowercase all text with Natural Language Toolkit (version 3.6.2).^22^ Term frequency-inverse document frequency (tf-idf) is calculated for the top 400 words with the highest term frequencies in the training set, followed by vector normalization.^23^^,^^24^ The features for the classifiers are:

1. 4 position indicators obtained from the OCR,
2. page number,
3. floating-point representation of the numeric value, and
4. tf-idf of the top 400 terms.

We evaluate well-established machine learning classifiers including Logistic Regression, Ridge Regression, Lasso Regression, Support Vector Machine, k-Nearest Neighbor, Naïve Bayes, and Random Forest. All models are implemented in *Scikit-learn* (version 0.24.2).^25^

### Deep learning-based sequence models

**Figure 3.**
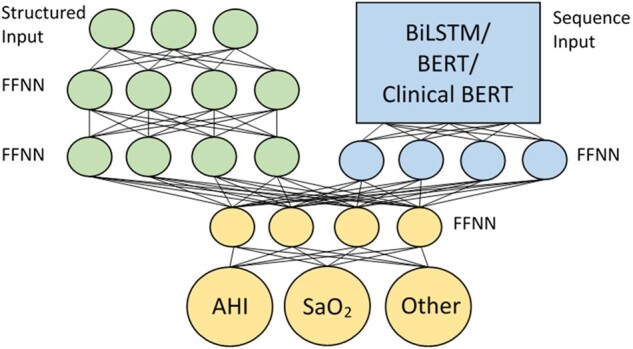
Parent neural network architecture. The structured input branch (top-left) takes in position indicators, page number, and numeric value. The sequence input branch (top-right) takes in encoded segments, processed by specific deep learning architectures, and flattened to remove time steps. The classifier layers (bottom) connect the structured input branch (green) and sequence input branch (blue) and make predictions.

In recent years, deep learning adoption in clinical NLP has grown substantially.^26^ To evaluate the efficacy of these models for our task, we evaluate Bidirectional Long short-term memory (BiLSTM), Bidirectional Encoder Representations from Transformers (BERT),^6^ and the continuously pretrained BERT using EHR data (ClinicalBERT).^27^ All deep learning models are evaluated as a component of a parent neural network architecture shown in Figure 3. The network includes an input branch for the structured features (position indicators, page number, numeric value in float). The inputs are batch-normalized,^28^ with 2 layers of feed-forward neural network (FFNN) with 100 neurons and a 20% dropout rate in each layer. The network also includes an input branch for segments (sequences). The sequence features input with a maximum sequence length of 32 tokens, is encoded, processed (with BiLSTM, BERT, or ClinicalBERT), flattened, and passed to an FFNN. The structured and sequence input branches are concatenated and fully connected to the classifier layers which includes a FFNN with 200 neurons and a 20% dropout rate, followed by the output layer with a sigmoid activation function. The outputs are multinomial with probabilities for the 3 categories: “AHI,” “SaO_2_,” and “Other.” All deep learning models are constructed with *TensorFlow* (version 2.2.0)^29^ and *Keras* (2.4.3).^30^

For BiLSTM, we use the word2vec^31^ embedding implemented with *Gensim* (version 4.0.1)^32^ and pretrained on the training set using Continuous bag-of-word (CBOW).^33^ We apply an embedding dimension of 100, then input the embedded word vectors to the model through 2 layers of BiLSTM where the second layer’s last hidden state is fed to the classifier layers.

We use the uncased BERT-base model^34^ in the *transformers* library (version 4.6.1)^35^ with TensorFlow. The segments are tokenized and embedded with WordPiece embedding^36^ before input into the BERT model. We flatten the outputs from BERT (a vector of 768 dimensions for each of the 32 input tokens) and pass them to a FFNN, followed by the classifier layers.

**Figure 4.**
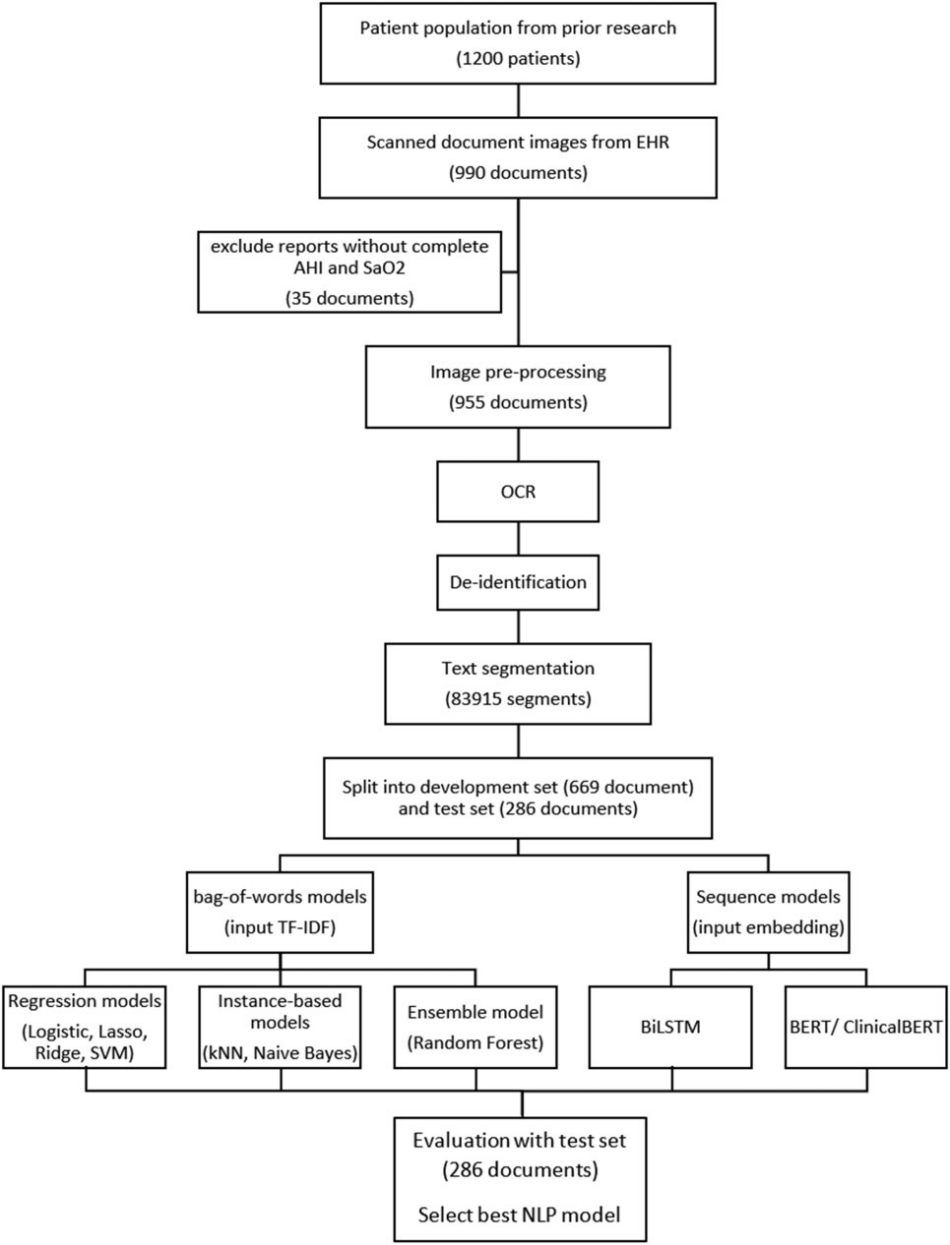
Data pipeline flowchart.

For ClinicalBERT, we use Bio_ClinicalBERT^37^ in the *transformers* library through *PyTorch* (version 1.9.0)^38^ and convert it to a TensorFlow model. The methodology flowchart is shown in Figure 4.

### Model training and evaluation

To examine the NLP models, we split the reports into a 70% (*N *=* *669) development set and a 30% test set (*N *=* *286). For the bag-of-words models, we performed a 5-fold cross-validation using the development set to search for an optimal parameter set that maximizes the validation accuracy. We then retrained each model with the entire development set given the optimal parameters. For the deep learning-based sequence models, due to the high computation, we further split the 70% development set with a 6:1 ratio into a training set (*N *=* *574) and a validation set (*N *=* *95). We saved checkpoints after each epoch and used the validation set to select the best checkpoint as our final model, based on cross-entropy loss. The BiLSTM model was trained using a batch size of 64, with Adam optimization with a learning rate of 2e−4 for 100 epochs. BERT and ClinicalBERT were fine-tuned using a batch size of 64, with Adam optimization with a learning rate of 2e−6 for 100 epochs.

After training, the final models were evaluated with the test set. We evaluated at the segment level using recall, precision, and the area under the receiver operating characteristic curve (AUROC) for AHI and SaO_2_. To better assess our final goal for information extraction, we also evaluated it at the document level. The numeric value in a document with the highest probability for AHI (or SaO_2_) was selected to represent the document. We define document accuracy as:

$$\mathrm{Document}\;\mathrm{accuracy}=\frac{\#\;\mathrm{of}\;\mathrm{documents}\;\mathrm{correctly}\;\mathrm{extracted}}{\#\;\mathrm{of}\;\mathrm{documents}\;\mathrm{in}\;\mathrm{test}\;\mathrm{set}}.$$

We performed DeLong’s test^39^ for comparing AUROC, and the chi-squared test for comparing document accuracy among the models. Family-wise error rates were adjusted using the Bonferroni procedure.

### Training set size effect analysis

To assess the effect of training set size on model performance, our second experiment focused on subsets of the training set. We independently sampled from the training set (*N *=* *574) and built subsets of 10, 25, 50, and 100 reports. We used each subset to train the BiLSTM, BERT, and ClinicalBERT models and used the validation set (*N *=* *95) to select final models based on cross-entropy loss. The final models were evaluated with the test set (*N *=* *286).

### Stand-alone validation analyses

To explore the impact of image preprocessing on the final performance, we examined 6 different image preprocessing methods: (1) gray-scale (baseline), (2) gray-scale + dilate/erode, (3) gray-scale+increase contrast by 20%, (4) gray-scale+increase contrast by 60%, (5) gray-scale+dilate/erode+increase contrast by 20% (our proposed method), and (6) gray-scale+dilate/erode+increase contrast by 60%. Figure 1 shows the output images on a scanned report. For each preprocessing method, OCR was performed followed by ClinicalBERT to evaluate performance.

Our proposed sequence models involved both structured and sequence features. To evaluate the contribution of structured features, we examined an architecture with only the sequence input branch (excluding the structured input branch). ClinicalBERT was used followed by the classifier layers.

## RESULTS

**Figure 5.**
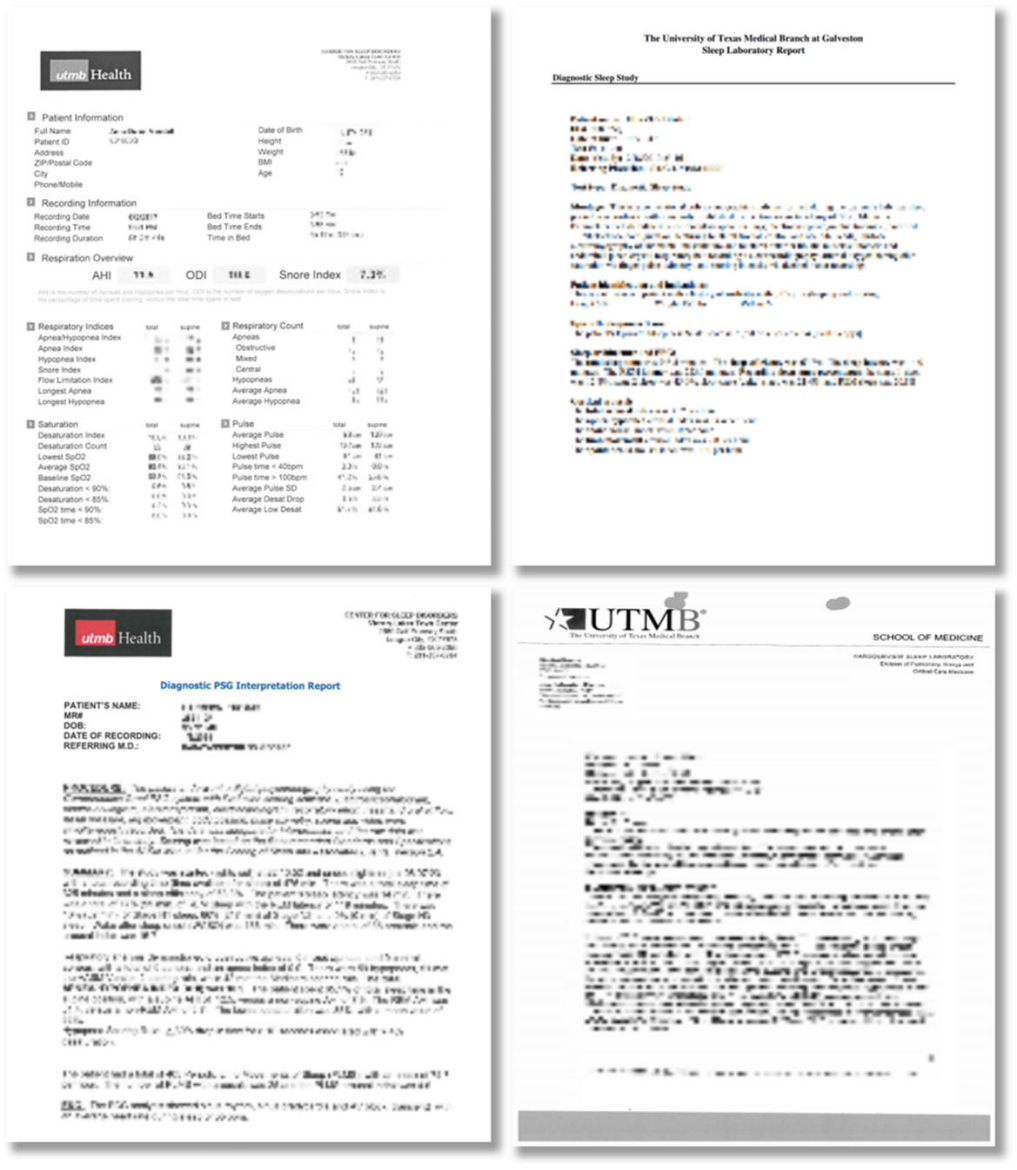
Collection of scanned sleep study reports. The images have been intentionally blurred, their purpose here is to provide a sense of the overall structure and consistency (and lack thereof) between scanned documents.

The sleep study reports were generated by different laboratories in various structures and layouts (Figure 5). From visual inspection of the original documents, most findings were reported in narratives in the printed text. The reports also contained images (eg, hospital logos, figures, and plots), tables, and handwriting. There were physician signatures and handwritten notes on the edges of some reports. Several reports from 1 laboratory have a similar structure of paragraphs and sentences, indicating a likelihood of templates being used.

The post-OCR inspection shows that most printed text, either in paragraphs or in tables, could be located. However, figures and handwriting added complexity. We noticed that in some reports, parts of images were considered text. Also, as reported in previous studies,^1^^,^^10^ we noticed some misspelled words in the outputs. For example, the letter “I” was recognized as “!” or “)”.

The scanned reports had a median of 2 pages (Q1–Q3* *=* *[2, 4], range* *=* *[1, 29]) and a median of 44 numeric values (Q1–Q3* *=* *[38, 106]) per page. About 52.8% of the reports had multiple numeric values that were labeled as AHI (median = 2, Q1–Q3* *=* *[1, 2]); 45.9% of the reports had multiple numeric values that were labeled as SaO_2_ (median = 1, Q1–Q3* *=* *[1, 2]) (Table 2). The AHI value has an average of 34.9 (Std Dev = 31.3, median = 24.4, Q1–Q3* *=* *[11.5, 48.9]). The SaO_2_ value has an average of 76.5 (Std Dev = 15.6, median = 80, Q1–Q3* *=* *[73.0, 85.8]).

**Table 2.** Summary of data and labels

	PDF documents		OCR outputs			
	Reports	Pages	Numeric values	Instances of AHI	Instances of SaO_2_	Instances of other
Entire data set	955	2988	83 915	1904	1698	80 313
development set	669	2031	56 839	1323	1146	54 370
Test set	286	957	27 076	581	552	25 943

**Figure 6.**
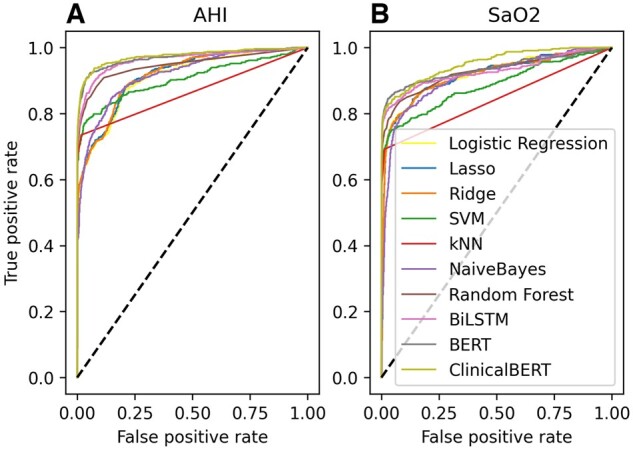
ROC curve for each classifier.

In our first experiment, the deep learning-based sequence models in general performed better than the bag-of-word models. For extraction of AHI, most bag-of-words models had high segment-level precision (0.4367–0.9865) that were close to sequence models (0.8803–0.9843). But sequence models had much higher recalls (0.6454–0.7470) compared to bag-of-words models (from 0.4802 to 0.6713). BERT and ClinicalBERT showed the highest F1 score of 0.8082 and 0.8126, and the highest AUROC of 0.9705 and 0.9743, respectively. At the document level, the best bag-of-word models, kNN, and Random Forest had around 93.5% accuracy while BERT and ClinicalBERT reached 94%–95% accuracy (Table 3 and Figure 6).

**Table 3.** Evaluation of different classifiers

	Classifier	Segment-level				Document-level
		Recall	Precision	F1	AUROC (95% CI)	Accuracy (95% CI)
AHI						
Bag-of-word models	LR	0.4819	0.8383	0.612	0.9093 (0.8932–0.9254)	87.41 (83.57–91.26)
	LASSO (L1)	0.4819	0.8889	0.625	0.9169 (0.9014–0.9325)	89.16 (85.56–92.76)
	Ridge (L2)	0.4802	0.8429	0.6118	0.9176 (0.9021–0.9331)	87.41 (83.57–91.26)
	SVM	0.6093	0.9752	0.75	0.9050 (0.8886–0.9215)	93.01 (90.05–95.96)
	kNN	0.6713	0.8534	0.7514	0.8644 (0.8454–0.8834)	93.57 (90.36–96.78)
	NaiveBayes	0.5577	0.4367	0.4898	0.9179 (0.9024–0.9334)	75.87 (70.92–80.83)
	Random Forest	0.6299	0.9865	0.7689	0.9476 (0.9350–0.9603)	93.71 (90.89–96.52)
Sequence models	BiLSTM	0.6454	0.9843	0.7796	0.9637 (0.9530–0.9743)	94.06 (91.32–96.80)
	BERT	0.747	0.8803	0.8082	0.9705 (0.9609–0.9802)	**95.10 (92.60–97.61)**
	ClinicalBERT	0.7315	0.914	**0.8126**	**0.9743 (0.9652–0.9833)**	94.76 (92.17–97.34)
SaO_2_						
Bag-of-word models	LR	0.567	0.4914	0.5265	0.9153 (0.8992–0.9314)	82.87 (78.50–87.23)
	LASSO (L1)	0.538	0.5103	0.5238	0.9151 (0.8990–0.9312)	84.62 (80.43–88.80)
	Ridge (L2)	0.5543	0.4904	0.5204	0.9143 (0.8981–0.9305)	83.22 (78.89–87.55)
	SVM	0.6105	0.9133	0.7318	0.8860 (0.8678–0.9042)	87.76 (83.96–91.56)
	kNN	0.587	0.8663	0.6998	0.8429 (0.8223–0.8634)	87.86 (83.84–91.88)
	NaiveBayes	0.6322	0.2705	0.3789	0.9082 (0.8915–0.9248)	51.75 (45.96–57.54)
	Random Forest	0.6087	0.9307	0.736	0.9264 (0.9113–0.9415)	89.51 (85.96–93.06)
Sequence models	BiLSTM	0.6739	0.9051	0.7726	0.9274 (0.9123–0.9424)	**91.61 (88.40–94.82)**
	BERT	0.7319	0.8651	**0.7929**	0.9358 (0.9215–0.9500)	**91.61 (88.40–94.82)**
	ClinicalBERT	0.683	0.8871	0.7718	**0.9523 (0.9398–0.9647)**	**91.61 (88.40–94.82)**

*Note*: Logistic Regression does not apply penalty; Lasso regression has L1 penalty (λ = 0.01); Ridge has L2 penalty (λ = 0.01). Support Vector Machine uses a polynomial kernel. kNN uses *k* = 3. NaiveBayes classifier uses alpha = 0.5. BiLSTM uses Word2Vec model for embedding pretrained on the training set with CBOW, input vector of 100 dimensions. BERT and ClinicalBERT are fine-tuned for 100 epochs with sequence length 32, and batch size 64. We highlight the highest F1, AUROC, and Accuracy in bold.

For SaO_2_ extraction, we found similar patterns to AHI. Sequence models had a much higher segment-level recall (0.6739–0.7319). ClinicalBERT achieved the highest AUROC of 0.9523. At the document level, sequence models had higher accuracy of 91.61% while bag-of-words model accuracy ranged from 51.75% to 89.51% (Table 3).

Comparing among sequence models, for AHI extraction, ClinicalBERT had significantly higher AUROC than BiLSTM (*P *=* *.0008). For SaO_2_ extraction, ClinicalBERT achieved the highest AUROC and was significantly higher than BERT (*P* = .0029) and BiLSTM (*P *<* *.0001). We did not see any significant document accuracy differences given the limited sample size in the test set (Table 4).

**Table 4.** Comparing ClinicalBERT with BERT, BiLSTM, and Random Forest

Adjusted *P*-value	ClinicalBERT vs BERT		ClinicalBERT vs BiLSTM		ClinicalBERT vs Random Forest	
	AHI	SaO_2_	AHI	SaO_2_	AHI	SaO_2_
AUROC	0.4528	**0.0029**	**0.0008**	**<0.0001**	**<0.0001**	**<0.0001**
Document accuracy	1.0000	1.0000	1.0000	1.0000	1.0000	1.0000

*Note*: AUROC was pair-wisely compared with DeLong’s test. Document accuracy was pair-wisely compared with the chi-squared test. All *P*-values are corrected with the Bonferroni procedure. We highlight statistically significant *P*-values in bold.

Summarizing the first experiment, our data pipeline with ClinicalBERT as the NLP model achieves the best performance for AHI extraction (AUROC of 0.9743, 94.76% document accuracy) and SaO_2_ extraction (AUROC of 0.9523, 91.61% document accuracy).

**Figure 7.**
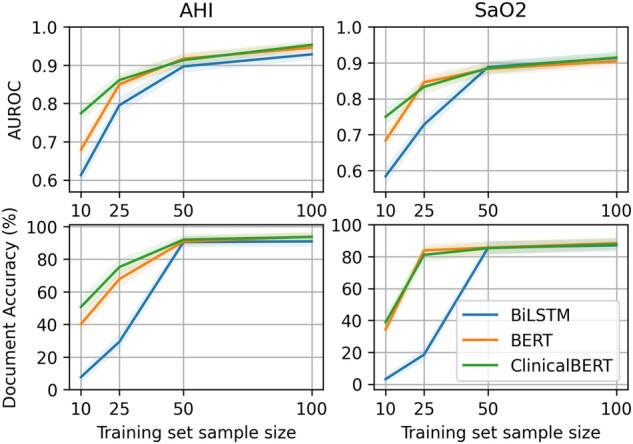
Evaluation of effects of training set size.

In our second experiment, we examined the effect of different training set sizes to reflect real-world conditions where large amounts of training data are often unavailable. For AHI extraction, ClinicalBERT reached an AUROC of 0.8612 and a document accuracy of 75.18% training on only 25 reports. With the same sample size, BERT had an AUROC of 0.8501 and a document accuracy of 67.83%. BiLSTM had an AUROC of 0.7954 and a document accuracy of 29.37%. When the sample size was 50 reports, the 3 deep learning-based models had a similar performance of around 0.9 of AUROC and 90% document accuracy. For SaO_2_ extraction, the ClinicalBERT and BERT had similar performances. Trained on 25 reports, they achieved an AUROC of 0.8333 and 0.8458, and a document accuracy of 81.12% and 83.92%, respectively, while BiLSTM had an AUROC of 0.7279 and a document accuracy of 18.53%. With 50 reports as the training set, all 3 models achieved AUROC of 0.88 and 85% document accuracy (Figure 7). Summarizing the second experiment, ClinicalBERT performs better with less training data. However, with at least 50 reports in the training set, all 3 models perform similarly well.

As a stand-alone validation analysis, we evaluated different image preprocessing methods followed by ClinicalBERT. The results showed that 1 iteration of dilating and eroding with an increased contrast of 20% resulted in the best performance for AHI extraction and second-best performance for SaO_2_ in AUROC (Table 5).

**Table 5.** Comparison of different image preprocessing methods

	Image preprocessing	Segment-level						Document-level
		Recall	Precision			F1	AUROC (95% CI)	Accuracy (95% CI)
AHI	Gray scale	0.7187		0.9249	0.8089		0.9699 (0.9601–0.9796)	95.45 (93.04–97.87)
	Gray scale+dilate and erode	0.6961		0.9687	0.81		0.9679 (0.9573–0.9784)	94.41 (91.74–97.07)
	Gray scale+contrast 20%	0.7126		0.9324	0.8078		0.9705 (0.9609–0.9802)	94.06 (91.32–96.80)
	Gray scale+contrast 60%	0.7268		0.9216	0.8127		0.9692 (0.9593–0.9790)	95.45 (93.04–97.87)
	Gray scale+dilate and erode+contrast 20%	0.7315		0.914	0.8126		**0.9743 (0.9652–0.9833)**	94.76 (92.17–97.34)
	Gray scale+dilate and erode+contrast 60%	0.7268		0.9216	0.8172		0.9715 (0.9620–0.9810)	**95.80 (93.48–98.13)**
SaO_2_	Gray scale	0.7258		0.8617	0.7879		0.9334 (0.9190–0.9478)	91.61 (88.40–94.82)
	Gray scale+dilate and erode	0.7427		0.8819	0.8063		**0.9620 (0.9504–0.9736)**	90.21 (86.77–93.65)
	Gray scale+contrast 20%	0.6957		0.8889	0.7805		0.9431 (0.9296–0.9566)	91.26 (87.99–94.53)
	Gray scale+contrast 60%	0.6863		0.8671	0.7662		0.9495 (0.9366–0.9623)	91.61 (88.40–94.82)
	Gray scale+dilate and erode+contrast 20%	0.683		0.8871	0.7718		0.9523 (0.9398–0.9647)	91.61 (88.40–94.82)
	Gray scale+dilate and erode+contrast 60%	0.6863		0.8671	0.7684		0.9486 (0.9356–0.9616)	**91.96 (88.81–95.11)**

*Note*: Each image preprocessing method was followed by fine-tuning a downstream  ClinicalBERT. We highlighted the highest AUROC and Accuracy in bold.

We also evaluated different sequence model architectures with and without structured inputs using ClinicalBERT. The results show adding structured input significantly improves AUROC by 0.0043 (*P* = .0092) for AHI, and 0.0107 (*P* = .0123) for SaO_2._ The document accuracy increases by 0.35% for AHI and 0.7% for SaO_2_ (Table 6).

**Table 6.** Comparison of different sequence model architectures

	Model architecture	Segment-level					Document-level	
		Recall	Precision	F1	AUROC (95% CI)	*P*-value	Accuracy (95% CI)	*P*-value
AHI	Sequence input	0.7522	0.8723	0.8078	0.9703 (0.9606–0.9800)	**.0092**	94.41 (91.74–97.07)	1.0000
	Sequence input+structured input	0.7315	0.914	0.8126	**0.9743 (0.9652–0.9833)**		**94.76 (92.17–97.34)**	
SaO_2_	Sequence input	0.692	0.8761	0.7733	0.9430 (0.9295–0.9565)	**.0123**	90.91 (87.58–94.24)	.8823
	Sequence input+structured input	0.683	0.8871	0.7718	**0.9523 (0.9398–0.9647)**		**91.61 (88.40–94.82)**	

*Note*: We highlighted the highest AUROC and Accuracy, and statistically significant *P*-value in bold.

## DISCUSSION

Our proposed data pipeline with appropriate image preprocessing and ClinicalBERT with structured input features showed excellent performance for extracting laboratory values from scanned documents. Our study design minimized the need for manual annotation. We utilized existing labeled reports, created annotated segments with automatic value-matching programs, trained NLP models, and proposed a data pipeline that extracted the needed variables with high performance. Our sample size experiment showed that BERT-based sequence models achieve 90% accuracy with a small training set, indicating flexibility for trading a small degree of performance for a significant reduction in annotation cost. Our data pipeline can be applied to information extraction of EHR documents, including laboratory reports, and clinical and imaging notes, in both scanned formats or machine-readable formats.

To analyze the difficulty of the NLP piece of the pipeline, we evaluated 7 bag-of-word models and 3 deep learning-based sequence models. Our evaluation showed ClinicalBERT achieves the best AUROC and document accuracy. This is consistent with a previous study that utilized ClinicalBERT for scanned document classification.^10^ The authors reported an accuracy of 97.3% for classifying clinically relevant documents versus not clinically relevant documents, while the Random Forest classifier achieved only 95.8%. They did not evaluate other deep learning models. Our evaluation covered a wider range of machine learning methods. We reported that BiLSTM, BERT, and ClinicalBERT performed better than bag-of-word models. Among bag-of-word models, Random Forest performed best. Our study is one of the first that evaluated deep learning-based NLP models for scanned document processing, though we also demonstrate that other aspects of the scanned document pipeline are important as well.

Though the open-source OCR library Tesseract has proven effective, appropriate image preprocessing is needed for realistic input.^15^ Few publications have focused on evaluating different image preprocessing methods for scanned medical documents. An earlier study that focused on evaluating OCR engines for scanned medical documents applied image preprocessing methods for overlapping-lines removal.^1^ In our documents, since the majority of the text was printed instead of hand-written, overlapping lines were not a significant issue. We referenced a recent study that utilized gray-scaling, erosion, and increasing contrast to improve image quality.^10^ Gray-scaling has been reported to improve Tesseract OCR performance. Dilation, erosion, and contrast are simple transformations that have a uniform effect when applied to images with different scanned quality, compared to complicated thresholding transformations. Thus, we chose these methods for data pipeline development. We believe our work is a practical starting point. To better validate the selection of image preprocessing methods, scanned documents with annotated text are needed. Some related studies included post-OCR text processing that involved spell-checking.^4^^,^^10^ We omitted this step considering the inconsistent abbreviations in our documents.

The presence of layout and word position is a unique feature in scanned documents compared to digitally stored text in EHR. In the scanned sleep study reports, there was often an organization logo on the top left, a title line in the middle, and a facility name and contact information on the top right. Important findings were often placed in obvious positions, for example, in the upper part of the first page. We noticed some laboratories have developed reporting templates. This also contributes to the consistency of word positioning. Therefore, when developing the data pipeline, we utilized the word positions and page numbers as additional information. Our validation analysis found that adding structured inputs including word position in pixels, page number, and numeric value could significantly boost model performances (ClinicalBERT alone: AHI AUROC = 0.9703; ClinicalBERT with structured input: SaO_2_ AUROC = 0.9743, *P* = .0092). To our knowledge, the use of word position and layout in NLP models has not been reported. We propose this is a new direction for optimizing NLP model performances.

Although AHI and SaO_2_ were often reported in writing, some reports presented them in tables. Our text segmentation process only captures right and left word sequences around the candidate numbers and does not incorporate words above or underneath them. Thus, table column names were not captured. Manually reviewing the cases that failed to be recognized by the model, a high proportion of them was from tables. Unfortunately, there is limited research about processing natural language in tables. Future studies are needed to resolve this problem.

Our study does have some limitations. First, the secondarily selected data’s cohort selection criterion emphasized patients with sleep disorders instead of all sleep study patients. However, we believe patient demographics and medical history would not significantly bias our findings. Besides, due to the current insurance reimbursement environment, having a sleep disorder diagnosis is often a prerequisite for reimbursement of a sleep study. Second, the human chart review was performed at the document level. To handle this, we matched AHI and SaO_2_ values and label all exact-matched numbers in the text as targets. Thus, numbers that by chance had the same value as a target would be mislabeled. This is possible, though unlikely, that some other measurements (eg, Apnea index, snore index) have overlapping reference ranges.

## CONCLUSION

This study examined the common workflow for scanned document information extraction and evaluated the interplay of each method: image preprocessing, OCR, and NLP. We show that dilation/erosion, increasing contrast, word-layout information, and advanced deep-learning improves the information extraction performance from scanned sleep study reports. Our results fill the gap in the field of EHR-scanned documents processing by providing a thorough evaluation of each moving part.

## FUNDING

This work was supported by a pilot grant of Biostatistics, Epidemiology and Research Design under UL1TR001439 from the National Center for Advancing Translation Sciences (NCATS), as well as award R21EB029575 from the National Institute of Biomedical Imaging and Bioengineering (NIBIB).

## AUTHOR CONTRIBUTIONS

EH conceived the study. EH and KR designed the study. EH and IM performed initial image processing. EH conducted the experiments. RS aided with data annotation and provided clinical expertise. YFK provided administrative support. EH and KR wrote the initial manuscript. All authors read and approved the final manuscript.

## CONFLICT OF INTEREST STATEMENT

None declared.

## SOFTWARE AVAILABILITY

Our source code is available: https://github.com/daviden1013/Scanned_sleep_study_info_extraction.

## DATA AVAILABILITY

The data underlying this article cannot be shared publicly due to data privacy concerns. Data may be shared under privacy approval and data use agreement with the originating institution.
